# Changes in adrenergic and cholinergic receptors on human cerebral vessels. Implications for new therapeutic strategies in Alzheimer's Disease

**DOI:** 10.1002/alz70855_102608

**Published:** 2025-12-23

**Authors:** Roxana O. O Carare

**Affiliations:** ^1^ University of Southampton, Southampton, Hampshire, United Kingdom

## Abstract

**Background:**

Sporadic cerebral amyloid angiopathy (CAA) occurs as a result of a failure of Intramural Periarterial Drainage (IPAD) of amyloid‐beta (Aβ) along the walls of capillaries and arteries. The motive force for IPAD is provided by the spontaneous contractions of cerebrovascular smooth muscle cells (CVSMC that receive adrenergic and cholinergic innervation from Locus Coeruleus and Nucleus Basalis of Meynert, compromised very early in Alzheimer's disease (AD). Here we tested the hypothesis that adrenergic and cholinergic receptors are downregulated in parenchymal and leptomeningeal arteries with CAA and AD.

**Method:**

Sections of postmortem brains from young, old and CAA‐AD cases from the Newcastle and Edinburgh Brain Banks UK were used for immunohistochemistry for muscarinic (M1, M2, M3), nicotinic (α7 nicotinic acetylcholine receptor) and adrenergic (α1b/2a/2b) receptors. The percentage area immunostained for each receptor was imaged and analysed (T‐test).

**Result:**

Cholinergic receptors: There was a significant decrease in the expression of α7 nicotinic acetylcholine receptors in the parenchymal vessels of old vs young and a significant decrease of α7 nicotinic acetylcholine receptors in the leptomeningeal vessels of CAA vs old. There were no changes in the expression of M1, M2 between the different groups and M3 was not present on blood vessels. Adrenergic receptors: There was a significant decrease in the expression of parenchymal α1b adrenergic receptors in old vs young and a significant decrease in the expression of leptomeningeal α2a and α2b adrenergic receptors in leptomeningeal vessels of CAA vs old.

**Conclusion:**

As IPAD is dependent on the integrity of the vascular smooth muscle cells, to maintain the integrity of IPAD with ageing, future therapeutic interventions could focus on agonists of α7 nicotinic acetylcholine and α adrenergic receptors.

